# A new Schiff base-fabricated pencil lead electrode for the efficient detection of copper, lead, and cadmium ions in aqueous media

**DOI:** 10.1039/d3ra02582a

**Published:** 2023-05-23

**Authors:** Abdelrahman S. Ahmed, Mahmoud Basseem I. Mohamed, Mahmoud A. Bedair, Adham A. El-Zomrawy, Moustafa F. Bakr

**Affiliations:** a Chemistry Department, Faculty of Science, Al-Azhar University 11884 Nasr City Cairo Egypt mahmoudbasseem@azhar.edu.eg; b Department of Chemistry, College of Science and Arts, University of Bisha P. O. Box 101 Al-Namas 61977 Saudi Arabia m_bedier@ub.edu.sa m_bedier@yahoo.com

## Abstract

Cu^2+^, Pb^2+^, and Cd^2+^ were individually and simultaneously determined using a novel and effective electroanalytical approach that has been devised and improved. Cyclic voltammetry was used to examine the electrochemical properties of the selected metals, and their individual and combined concentrations were determined by square wave voltammetry (SWV) using a modified pencil lead (PL) working electrode functionalized with a freshly synthesized Schiff base, 4-((2-hydroxy-5-((4-nitrophenyl)diazenyl)benzylidene)amino)benzoic acid (HDBA). In a buffer solution of 0.1 M tris–HCl, heavy metal concentrations were determined. To improve the experimental circumstances for determination, scan rate, pH, and their interactions with current were studied. At some concentration levels, the calibration graphs for the chosen metals were linear. The concentration of each metal was altered while the others remained unchanged for both the individual and simultaneous determination of these metals, and the devised approach was proven to be accurate, selective, and rapid.

## Abstract 2

Cu^2+^, Pb^2+^, and Cd^2+^ were individually and simultaneously determined using a novel and effective electroanalytical approach that has been devised and improved.

## Graphical abstract

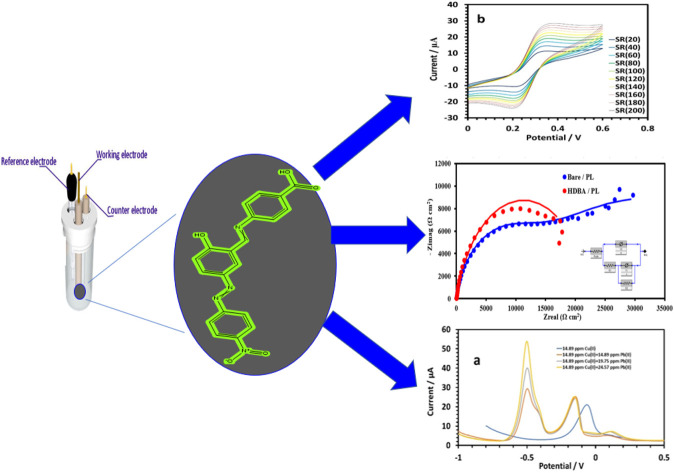

## 1 Introduction

Toxic heavy metals are released into the environment in ever-increasing quantities, endangering humanity as they build up in the soil and in human bodies due to their inability to disintegrate. They also pose a serious issue as pollutants in waste that is dumped into outflows. The cumulative properties and prolonged biological half-lives of these heavy metals make them potentially harmful to human health when exposed to them. Reactive oxygen species (ROS), in particular polyunsaturated lipids and proteins, can disrupt cell macromolecules as a result of metal toxicity.^1^ It is therefore important to detect and quantify heavy metals in soil and streams for health reasons.^2^ Spectroscopy,^3^ optical colorimetry,^4^ inductively coupled plasma^5^ mass spectrometry (ICP-MS),^6^ atomic absorption spectrometry (AAS),^7^ and fluorescence spectrometry^8^ are some of the analytical methods used today for heavy metal detection. However, they are expensive (they usually need expert operators and sophisticated equipment), therefore, electrochemical approaches have been researched as alternatives^9^ due to the ease of sample preparation and simpler procedures. In addition, electrochemical techniques for heavy metals analysis have certain advantages owing to their high sensitivity, precision, simplicity, and low cost.^10^ Schiff bases are chemical compounds formed when a primary amine reacts with an aldehyde or a ketone to form an imine.^11^ Schiff bases can be easily altered by selecting the right amine and substituting a carbonyl molecule, resulting in a wide range of structural diversity. Schiff bases are widely used in electrochemical applications owing to their characteristic structure and their ability to bind to electrode surfaces.^12–14^ The search for an optimum pencil graphite electrode (PGE) for the detection of trace heavy metals has exploded recently. Working electrodes made of carbon-based materials are often employed in electrochemical analysis^15^ owing to the ease with which they can be used to pre-treat surfaces. Given their convenience, time-saving nature, and compliance with green chemistry standards, disposable pencil-graphite electrodes (PGEs) are considered an excellent choice as working electrodes for both laboratory and on-site screening experiments. Pencil-graphite electrodes that have undergone electrochemical treatment also offer benefits for being small, affordable, easy to create, and simple to use. The graphite-based working electrodes are widely used in electrochemical analysis owing to its abundant porosity, high electrical conductivity, and large specific surface area.^16,17^ They also support rapid mass transport and fast electron transfer in electrochemical applications. It is crucial to be able to change the graphite in PGEs in just one step^6^ and this depends on the supramolecular interaction of the ions with a functional group on the electrode surface, which can allow Schiff base-modified electrodes to be able to acquire the target metal ions, and which has thus piqued research interest in recent years.^18^ In this study, we tried to achieve sensitive metal sensing by modifying a very cheap pencil lead (PL) electrode with the electrochemically modified Schiff base 4-((2-hydroxy-5-((4-nitrophenyl)diazenyl)benzylidene)amino)benzoic acid (HDBA), and termed this the HDBA/PL electrode. For this reason, we applied HDBA film to the PL electrode and performed numerous measurements. We investigated the PL electrode's surface area, the effects of pH, the voltammetry scan rate, and calculated the diffusion coefficient using cyclic voltammetry (CV). Additionally, individual metal detection by square wave voltammetry (SWV) was performed based on changes in the current from varying the concentration of specific metals, as well as the simultaneous determination of multiple metal ions, also by (SWV).^19^

## 2. Experimental

### 2.1. Chemicals and apparatus

The chemicals used in this study included 4-amino benzoic acid, tris–HCl buffer, methanol, dichloromethane, glacial acetic acid, CuSO_4_·5H_2_O, Pb(NO_3_)_2_, and Cd(NO_3_)_2_·4H_2_O. A Gamry REF 3000 potentiostat/galvanostat/Zra and Echem Analyst 7.8.2 program for data processing, fitting, and graphing were used for all electrochemical studies. The cell configuration used was a typical three-electrode configuration, with the HDBA-modified PL electrode utilized as the working electrode, and platinum wire and saturated Ag/AgCl utilized as the counter and reference electrodes, respectively.^20,21^. Bruker DMX-400 spectrometers running at 400 and 101 MHz were used to measure the ^1^H and ^13^C NMR spectra. An Agilent Cary 630 spectrometer was utilized to measure the FT-IR spectra. A 3520 pH meter was utilized to measure the pH levels (Jenway, UK). A Stuart melting point SMP30 instrument was used to measure the melting points.

### 2.2. Synthesis of HDBA

**Fig. 1:**
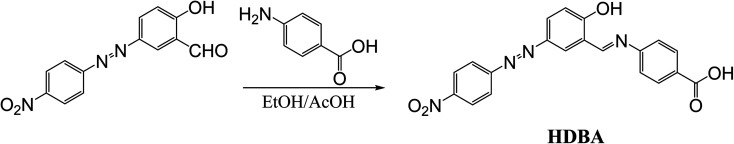
Synthetic route of the Schiff base (HDBA).

**Fig. 2:**
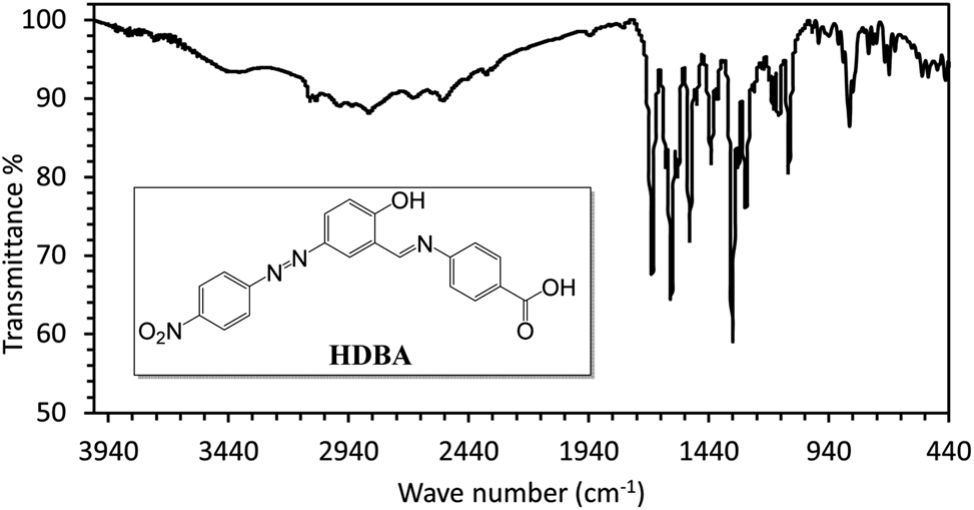
IR spectrum of HDBA.

**Fig. 3:**
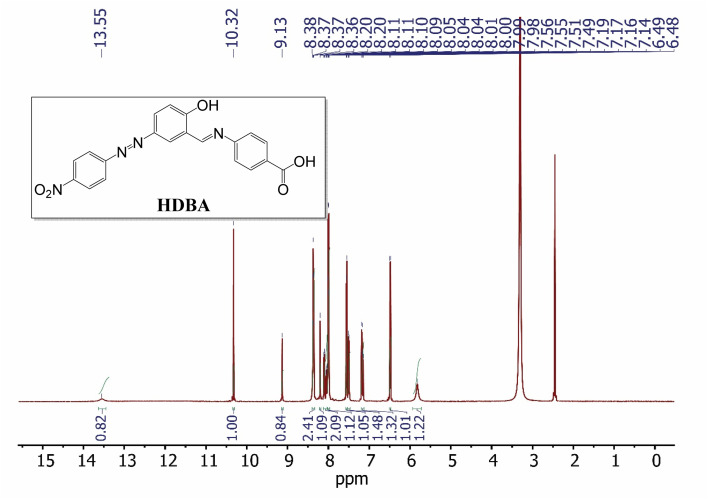
^1^H NMR spectrum of HDBA in DMSO-d_6_.

**Fig. 4:**
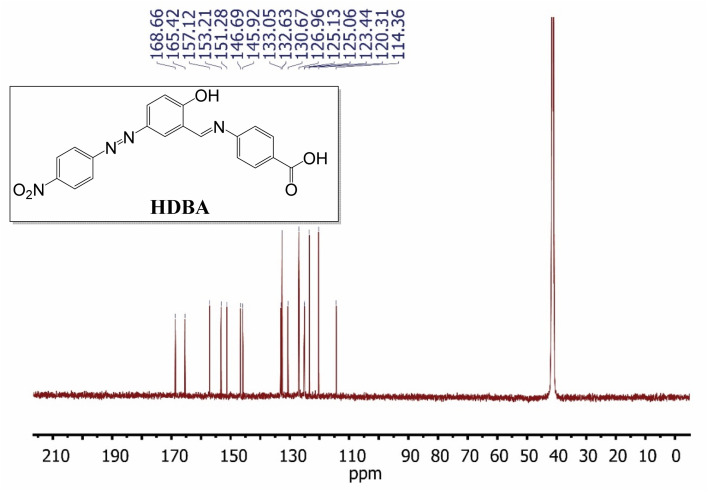
^13^C NMR spectrum of HDBA in DMSO-d_6_.

First, 2-hydroxy-5-((4-nitrophenyl)diazenyl)benzaldehyde (200 mmol)^22^ and 4-amino benzoic acid (200 mmol) were refluxed in ethanol for 3 h to afford a new Schiff base (HDBA), which was washed, dried, and recrystallized from ethanol to obtain a high-quality product in a 60–65% yield with a melting point of 250 °C (Fig. 1). IR, ^1^H-NMR, and ^13^C-NMR spectroscopy were used to determine the chemical structure of the synthesized Schiff base, as illustrated in Fig. 2, 3, and 4, respectively.^20^

The IR spectrum of HDBA displayed absorption bands at *V*_max_ = 3455 (O–H phenolic group), 3103 (C–H aromatic), 2853 (O–H, carboxylic group), 1676 (>C=O), 1620 (C=C), 1597 (C=N), and 1520, 1341 cm^−1^ (NO_2_ group).

^1^H-NMR (400 MHz, DMSO-d_6_, *δ* ppm): 5.82 (s, 1H), 6.49 (d, *J* = 8.7 Hz, 1H), 7.17 (dd, *J* = 12.4, 8.9 Hz, 1H), 7.50 (d, *J* = 8.2 Hz, 1H), 7.56 (d, *J* = 8.6 Hz, 1H), 8.00 (dd, *J* = 8.9, 2.4 Hz, 1H), 8.07 (ddd, *J* = 28.0, 8.8, 2.3 Hz, 2H), 8.20 (d, *J* = 2.6 Hz, 1H), 8.37 (dd, *J* = 8.7, 5.2 Hz, 2H), 9.13 (s, 1H), 10.32 (s, 1H), 13.55 (s, 1H).

^13^C-NMR (126 MHz, DMSO-d_6_, *δ*, ppm): 114.36, 120.31, 123.44, 125.06, 125.13, 126.96, 130.67, 132.63, 133.05, 145.92, 146.69, 151.28, 153.21, 157.12, 165.42, 168.66.

### 2.3. Construction of the HDBA/PL electrode

PL with a diameter of 0.9 mm and a length of 6 cm was used to create the working electrode. The surface wax was first removed using white print paper, and 1 cm of the PL was then dipped into a solution containing DCM and 1 : 1 HDBA + PVC to electrochemically synthesize HDBA film on the PL. To remove any potential impurities, the as-fabricated HDBA/PL electrode was soaked in 0.1 M tris–HCl solution for 15 min before being placed in a desiccator at room temperature for further testing.^19^

### 2.4. Electrochemical measurements

Utilizing cyclic voltammetry (CV) and square wave voltammetry (SWV), electrochemical tests were conducted. In 0.1 M tris–HCl, the potential was examined by CV analysis. The SWV parameters were: frequency of 12.5 Hz, 50 ms pulse width, 2 mV scan increment, and 50 mV pulse height. First, 200 ppm metal ions in solutions were generated at pH 2–10. Then, the relation between the potential and current was established and the pH was selected with the largest current that would impact the scan rate at a concentration of 50 ppm (CV). The relation among the current and various concentrations was established utilizing SWV as well.

## 3. Results and discussion

### 3.1. Characterization of HDBA

The IR spectrum of HDBA (Fig. 2) revealed multiple absorption peaks with distinctive characteristics. Absorption peaks were specifically identified at *V*_max_ = 3455 cm^−1^, corresponding to the O–H stretching of the phenolic group. Additional peaks at 3103 and 2853 cm^−1^ were noted corresponding to C–H stretching in aromatic and carboxylic groups, respectively. The peak at 1676 cm^−1^ revealed the presence of a carbonyl (>C=O) group, while the peaks at 1620 and 1597 cm^−1^ were attributed to the stretching vibrations of C=C and C=N bonds, respectively. The existence of the NO_2_ group was shown by the peaks at 1520 and 1341 cm^−1^.

The ^1^H-NMR spectrum of HDBA (Fig. 3) exhibited a number of proton-related signals. The proton signals between 5.82 and 8.73 ppm could be attributed to protons on the benzene ring. The 9.13 ppm singlet corresponded to the proton on the imine group, while the 10.32 ppm singlet was attributed to the proton on the phenolic group. Finally, the proton on the carboxylic acid group was indicated by the signal at 13.55 ppm.

The ^13^C-NMR spectrum of HDBA (Fig. 4) displayed several signals that were attributed to carbon atoms, notably the carbon atoms in the benzene ring, with signals between 120 and 140 ppm, in the azo group at 165.42 ppm, the C=N group at 157.12 ppm, and the carboxylic group at 186.66 ppm. These results were consistent with the expected chemical structure of the synthesized Schiff base.

### 3.2. Electrochemical measurements

#### 3.2.1. Electrochemical characterization of the HDBA/PL electrode using the standard potassium ferricyanide system

**Fig. 5:**
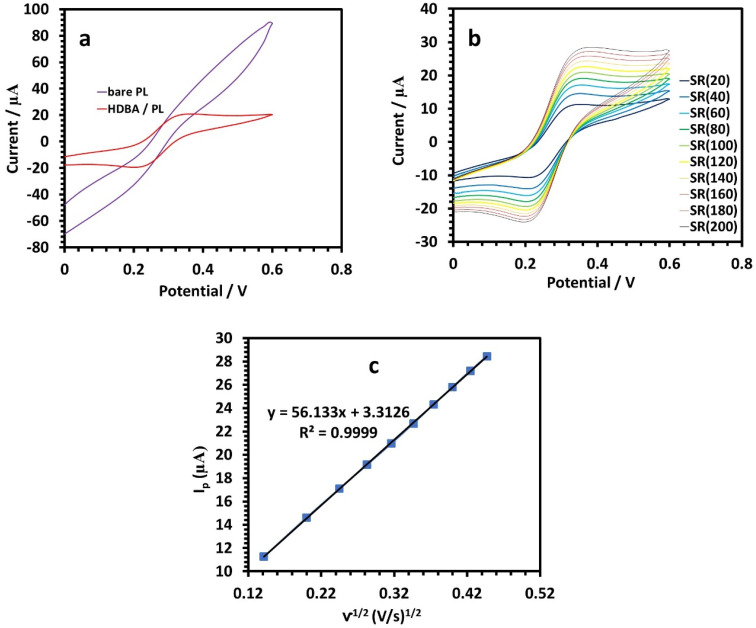
(a) CV curves of the bare PL and HDBA/PL electrodes in 10 mM [Fe(CN)_6_]^3−/4−^ in 0.1 M KCl at 100 mV s^−1^. (b) CV curves of the HDBA/PL electrode in 10 mM [Fe(CN)_6_]^3−/4−^ in 0.1 M KCl at scan rates from 20–200 mV s^−1^. (c) Plot of log *I*_p_*vs.* square root of scan rate. Potential *vs.* Ag/AgCl (3 M KCl).

The as-fabricated HDBA/PL electrode was electrochemically characterized using CV and a 10 mM ferricyanide redox probe dissolved in 0.1 M KCl. The findings showed that the addition of the Schiff base HDBA to the PL electrode increased the peak currents (Fig. 5a and b). The signal amplification was outstanding and was related to the increase in the electrode's electroactive surface area.^23^ To calculate the HDBA/PL electrode effective surface area for a reversible reaction, the Randles–Sevcik equation, was utilized:^24,25^

1 *i*_p_ = (2.69 × 10^5^)*n*^3/2^*AC*(*Dv*)^1/2^

where, *i*_p_ is the current maximum (Ampere), the constant 2.69 × 10^5^ has units of C mol^−1^ V^−1/2^, *n* denotes the number of transported electrons, *A* is the effective surface area of the HDBA/PL electrode, which was 0.06 mm^2^, and *D*, *C*, and *v* are the diffusion coefficient (cm^2^ s^−1^), the bulk concentration of the redox probe (mol cm^−3^), and the voltage sweep (V s^−1^), respectively. For K_3_Fe(CN)^6^, *n* = 1 and *D* = 7.6 × 10^−5^ cm^2^ s^−1^. Here, a linear association was found between the peak current *vs.* square root in the scan rate plot, indicating that the electrochemical kinetics was a diffusion-controlled reaction (Fig. 5c). With a correlation value of *R*^2^ = 0.9999, the slope of the plot was calculated to be 56.133. According to the findings, the HDBA/PL electrode is suitable for the electroanalysis of heavy metal ions.^26^

**Fig. 6:**
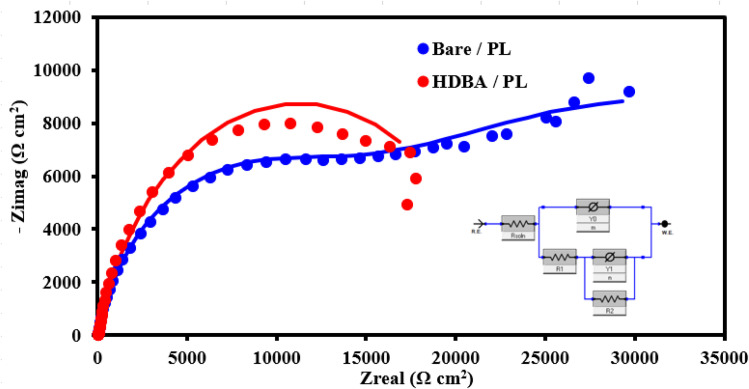
Typical Nyquist plots of the bare and HDBA/PL electrodes.

Next, electrochemical impedance spectroscopy (EIS) tests were done for the bare and HDBA electrodes in 10 mM ferricyanide redox probe dissolved in 0.1 M KCl in the frequency range of 0.1 Hz to 10^5^ Hz. The Nyquist spectra of the bare and HDBA/PL electrode are shown in Fig. 6. The electron transfer resistance *R*_CT_ was calculated from the Nyquist plot semicircles by fitting to the suitable equivalent circuit.^27,28^ The modified electrode had a smaller diameter, lower *R*_CT_, and higher conductivity than the bare electrode, whereby the value of *R*_CT_ for the bare electrode was 12.440 kΩ and the value of *R*_CT_ for the HDBA/PL electrode was 0.698 kΩ. Also, the bigger semicircle of the bare electrode demonstrated its greater interface impedance.^29,30^ The spectra were in good agreement with the outcomes of the CV experiments shown in Fig. 5a, demonstrating the modified electrode's increased conductivity.^31,32^

#### 3.2.2. Effect of pH

**Fig. 7:**
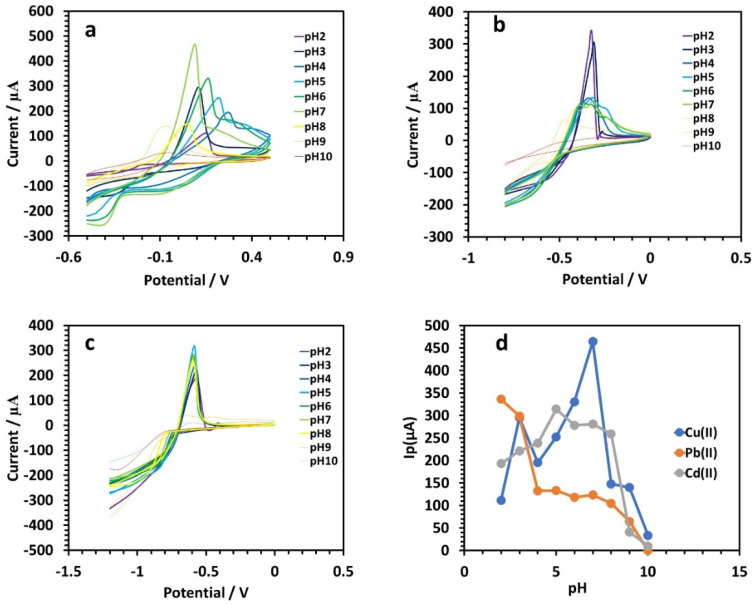
(a)–(c) CV curves of Cu, Pb, and Cd, respectively, at different pH values (200 ppm Cu(ii), Pb(ii), and Cd(ii)) in 0.1 M tris–HCl buffer at the HDBA/PL electrode. (d) pH dependence of *I*_p_ for Cu(ii), Pb(ii), and Cd(ii).

In 0.1 M tris–HCl buffer, the effect of Cu, Pb, and Cd(ii) on the maximum current was investigated.^33^ The results are given in Fig. 7a–c, showing the ideal pH for copper, lead, and cadmium determination was 6, 2, and 5, respectively. The height of the Cu, Pb, and Cd(ii) peak currents dropped when the acidity was higher or lower than the ideal pH value.^34^ The linear graphs of the peak potential's pH dependency are shown in Fig. 7d.

#### 3.2.3. Impact of the scan rate

**Fig. 8:**
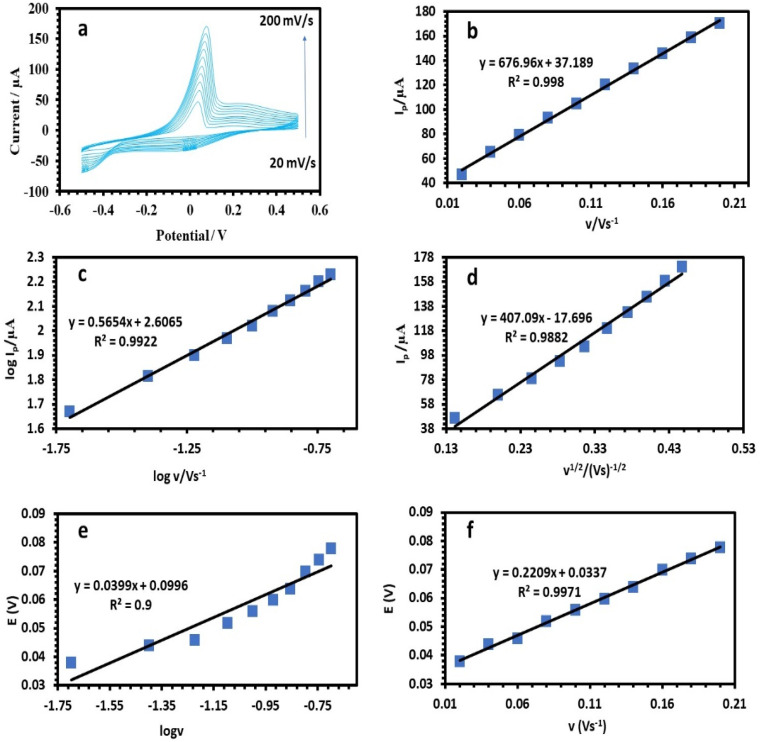
(a) Cyclic voltammetry responses of 50 ppm Cu(ii) solution at the HDBA/PL electrode in 0.1 M tris–HCl buffer solution pH 6 at different scan rates from 20–200 mV s^−1^. (b) Plots of peak currents *vs. υ*. (c) Plots of log peak currents *vs.* log *υ*. (d) Plots of peak currents *vs. υ*^1/2^. (e and f) Variation of the peak potential *vs.* log *υ* and *υ*, respectively.

**Fig. 9:**
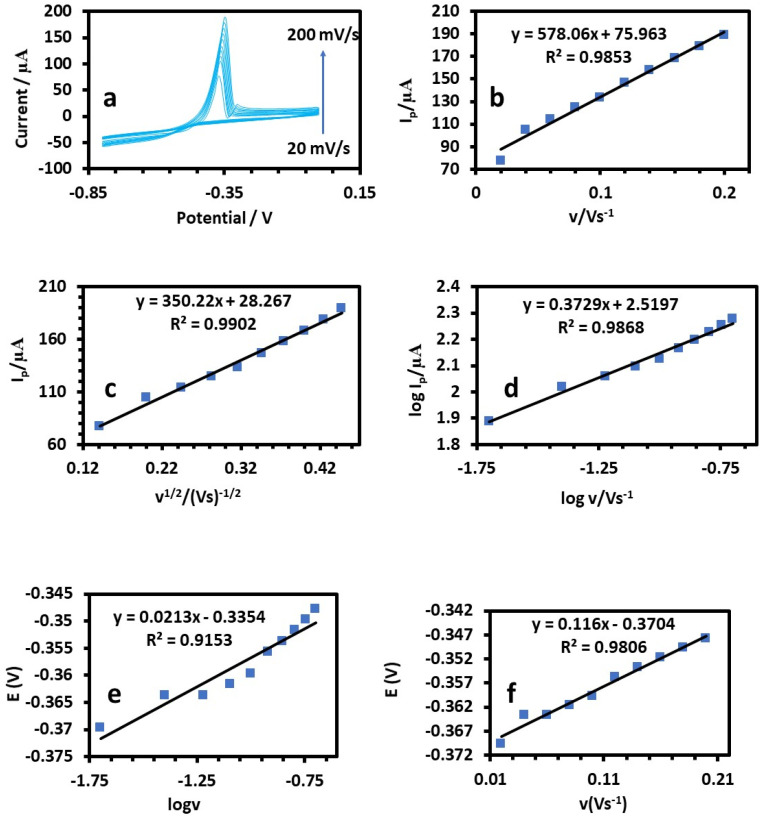
(a) Cyclic voltammetry responses of 50 ppm Pb(ii) solution at the HDBA/PL electrode in 0.1 M tris–HCl buffer solution pH 3 at different scan rates from 20–200 mV s^−1^. (b) Plots of peak currents *vs. υ*. (c) Plots of log peak currents *vs.* log *υ*. (d) Plots of peak currents *vs. υ*^1/2^. (e and f) Variation of the peak potential *vs.* log *υ* and *υ*, respectively.

**Fig. 10:**
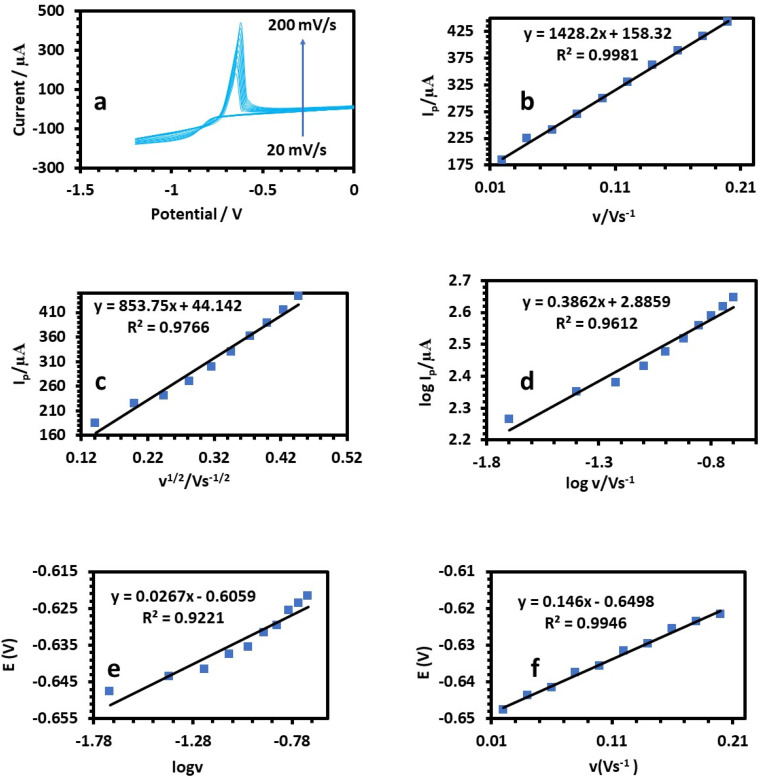
(a) Cyclic voltammetry responses of 50 ppm Cd(ii) solution at the HDBA/PL electrode in 0.1 M tris–HCl buffer solution pH 5 at different scan rates from 20–200 mV s^−1^. (b) Plots of peak currents *vs. υ*. (c) Plots of log peak currents *vs.* log *υ*. (d) Plots of peak currents *vs. υ*^1/2^. (e and f) Variation of the peak potential *vs.* log *υ* and *υ*, respectively.

The relation between the peak current and scan rate can allow estimating important electrochemical mechanism information.^33^ Therefore, CV was applied to determine how the scan rate affected the peak potential (*E*_p_) and peak current (*I*_p_), as shown in Fig. 8a, 9a, and 10a. It is known that *E*_p_ is independent of *v* and *vice versa* if the electrooxidation reaction is reversible.^35^Fig. 8a, 9a, and 10a show that as the scan rate increased, the anodic peak potential (*E*_p_) moved to a higher potential, suggesting that the electron transfer in the analyte electrooxidation was irreversible. According to Fig. 8a, 9a, and 10a, the anodic peak current (*I*_pa_) increased with the scan rate increasing from 0.02 to 0.2 V s^−1^, indicating that the electron transfer reaction took place.^36^ A reasonable estimate of the surface concentration of the electroactive species (*Γ*) can be made using the slope of the linear plot of *I versus v* in Fig. 8b, 9b, and 10b by using the following equation:^37^

2 *i*_p_ = (*n*^2^*F*^2^*νAΓ*)/4*RT*

A linear relationship between log *I*_p_ and log *v* was found (Fig. 8c, 9c, and 10c). For the current regulated only by diffusion, the slopes of 0.5654, 0.3729, and 0.3862 V s^−1^ in the figures were close to the value of 0.5 V s^−1^. Consequently, the *I versus v*^1/2^ curve was linear, most likely as a result of diffusion-controlled oxidation (Fig. 8d, 9d, and 10d). According to Laviron, the following equation defines *E*_p_ as an irreversible electrode process:3
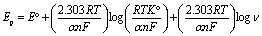
where *E*° denotes the formal oxidation and reduction potential, *n* denotes the number of transported electrons, *α* denotes the transfer coefficient, *k*° denotes the reaction's standard heterogeneous rate constant, and *v* denotes the scan rate. The computed (*k*°) values for Cu, Pb, and Cd at *T* = 298 K, *R* = 8.314 J K^−1^ mol^−1^, and *F* = 96 480 C mol^−1^ were 2596, 2450, and 3760, respectively (Table 1).^38^ The plots of *E* of the analytes *versus* log *v* are shown in Fig. 8e, 9e, and 10e. The Bard and Faulkner equation^39^ was used to get the value of *α*:4
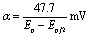
where *E*_p/2_ is the potential where the maximum current is cut in half, and based on these values of *α* was equal to 0.75, 0.81, and 0.9 for Cu, Pb, and Cd, respectively. Also, *n* for Cu, Pb, and Cd were also estimated to be 1.76–2, 1.78–2 and, 1.92–2, respectively (Table 1).^38,40^ According to Table 2, the peak potential favorably moved from 0.356 to 0.367 V when the scan rate was increased from 20 to 200 mV s^−1^ as a result of diminishing the diffusion layer growth in the electrode,^41^ and consequently, the estimated diffusion coefficient, *D*, increased linearly with the scan rate variation.^42^

**Table 1:** Parameters obtained from the Laviron equation

Metal ion	*α*	*n*	*K*°
Cu(ii)	0.75	1.76–2	2596
Pb(ii)	0.81	1.78–2	2450
Cd(ii)	0.90	1.92–2	3760

**Table 2:** Scan rate parameters of HDBA and their properties

Metal ion	Scan rate (mV s^−1^)	Anodic peak potential, *E*_pa_ (V)	Anodic peak current, *I*_pa_ (μA)	Square root of scan rate (*υ*^1/2^ s^−1/2^)	Diffusion coefficient (cm^2^ s^−1^)
Copper	20	0.037	46.74	0.141	13.10
	40	0.043	65.30	0.200	12.78
	60	0.045	79.07	0.244	12.50
	80	0.051	92.94	0.282	12.95
	100	0.053	104.9	0.316	13.20
	120	0.059	120	0.346	14.39
	140	0.063	133	0.374	15.15
	160	0.069	145.6	0.400	15.89
	180	0.073	158.6	0.424	16.76
	200	0.077	170.4	0.447	17.41
Lead	20	−0.369	77.39	0.141	63.21
	40	−0.363	104.6	0.200	57.74
	60	−0.363	114.3	0.244	45.96
	80	−0.361	124.9	0.282	41.16
	100	−0.359	133.4	0.316	37.56
	120	−0.355	146.8	0.346	37.91
	140	−0.353	157.8	0.374	37.54
	160	−0.351	168.3	0.400	37.37
	180	−0.349	178.9	0.424	37.53
	200	−0.347	189.1	0.447	37.74
Cadmium	20	−0.647	183.9	0.141	309.6
	40	−0.643	224.2	0.200	230.1
	60	−0.641	240	0.244	175.77
	80	−0.637	270	0.282	166.84
	100	−0.635	299	0.316	163.7
	120	−0.631	329.5	0.346	165.65
	140	−0.629	361.2	0.374	170.62
	160	−0.625	388.3	0.400	172.54
	180	−0.623	415.5	0.424	175.6
	200	−0.621	442.6	0.447	179.34

#### 3.2.4. Individual determination and calibration curves of the heavy metal ions

**Fig. 11:**
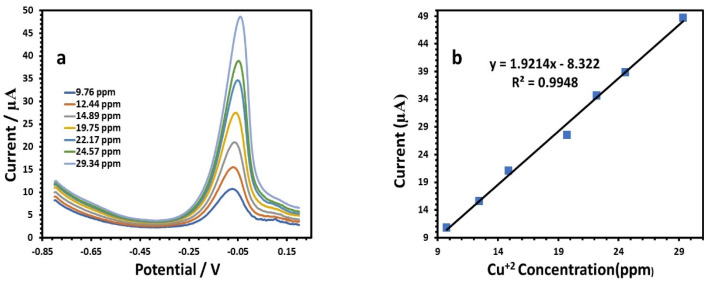
(a) SWV responses of the HDBA/PL electrode with successively adding different concentrations of Cu(ii) solution in 0.1 M tris–HCl buffer solution pH 6. (b) Calibration plot of the peak current toward the Cu(ii) concentration.

Fig. 11a depicts the respective square wave voltammetry (SWV) results for Cu(ii) at the modified electrode. The following is the linear equation for Cu(ii) at a concentration of 9.76 to 29.34 ppm:

5 *I* (μA), Cu = 1.9214*C* (ppm) − 8.322, (*r* = 0.9948)

**Fig. 12:**
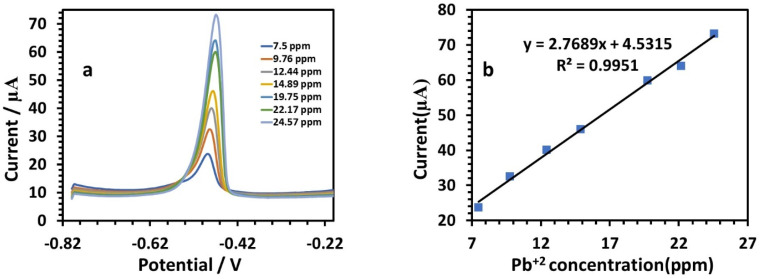
(a) SWV responses of the HDBA/PL electrode with successively adding different concentrations of Pb(ii) solution in 0.1 M tris–HCl buffer solution pH 3. (b) Calibration plot of the peak current toward the Pb(ii) concentration.

At a potential of approximately −0.09 V, the anodic behavior of Cu was visible, with a sensitivity of 1.9214 (Table 3). Fig. 12a illustrates the results for the determination of Pb(ii) at the modified electrode. According to the amount of Pb, the peak current increased gradually when the potential was about −0.49 V, where the anodic peaks could be observed.^39^ The SWV responses of the modified HDBA/PL electrode to Pb(ii) in the concentration range of 7.5 to 24.57 ppm (Table 3) are shown in Fig. 12b. The peak currents and concentrations were related by the following linear equation:

6 *I* (μA), Pb = 2.7689*C* (ppm) + 4.5315, (*r* = 0.9951)

**Table 3:** Calibration parameters for the individual determination of Cu(ii), Pb(ii), and Cd(ii) by HDBA

Metal ion	Cu(ii)	Pb(ii)	Cd(ii)
Linearity range (ppm)	9.76–29.34	7.5–24.57	7.5–24.57
Slope of the calibration plot	1.9214	2.7689	6.1955
Intercept	−8.322	4.5315	−20.892
Correlation coefficient (*r*)	0.9948	0.9951	0.999

**Fig. 13:**
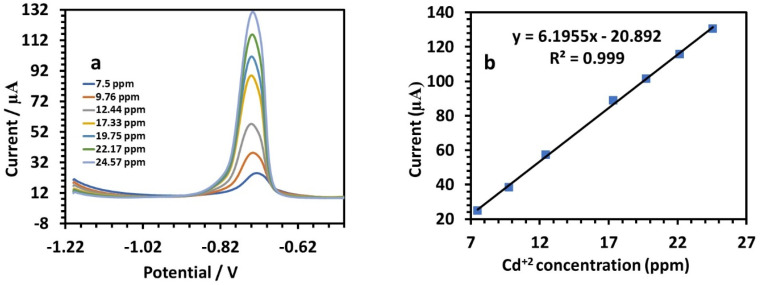
(a) SWV responses of the HDBA/PL electrode with successively adding different concentrations of Cd(ii) solution in 0.1 M tris–HCl buffer solution pH 5. (b) Calibration plot of the peak current toward the Cd(ii) concentration.

The sensitivity was 2.7689. Fig. 13a shows the SWV responses of the modified electrode to Cd(ii). The following is the linear equation for Cd(ii) at a concentration of 7.5 to 24.57 ppm (Fig. 12b):

7 *I* (μA), Cd = 6.1955*C* (ppm) − 20.892 (*r* = 0.999)

The sensitivity was 6.1955 (Table 3).^43^

#### 3.2.5. Simultaneous determination of the heavy meal ions

##### 3.2.5.1 Simultaneous determination of copper and lead

**Fig. 14:**
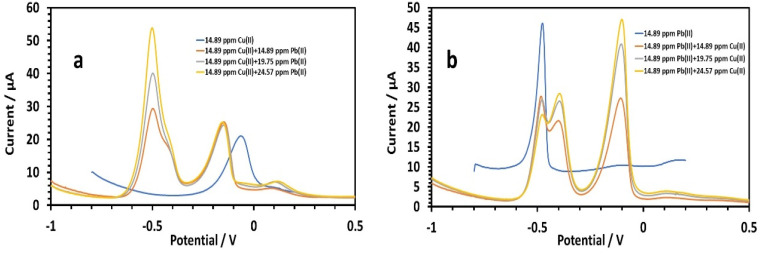
(a) Square wave voltammograms of HDBA/PL electrode solutions with different concentrations of Pb(ii) in the presence of a constant amount of Cu in 0.1 M tris–HCl buffer solution pH 6, (b) and with different concentrations of Cu(ii) in the presence of a constant amount of Pb(ii) in 0.1 M tris–HCl buffer solution pH 3.

To determine whether the presence of multiple cations has an effect, we tested the simultaneous detection of Cu^2+^ and Pb^2+^ in the same solution. First, the Cu^2+^ concentrations were set with varying the Pb^2+^ concentration, and *vice versa*^44^ (Fig. 14a and b). Lead's effect on 14.89 ppm copper in 0.1 M tris–HCl buffer was studied (Fig. 14a). The peak currents of Cu(ii) were slightly enhanced by the increasing lead concentration (14.89,19.75, and 24.57 ppm). The following is the linear equation for Pb(ii) with regard to concentrations of 14.89 to 24.57 ppm:^26^

8 *I* (μA), Pb = 2.5212*C* (ppm) − 8.66, (*r* = 0.9941)

The HDBA/PL electrode had a lower sensitivity to Pb(ii) when copper was present as 2.5212, and a higher sensitivity without copper of 2.77. The oxidation of Pb–Cu intermetallic compounds could be noted by the shoulder peaks near Pb. Furthermore, the peak currents of copper marginally decreased from −0.8 to −0.17 V. The impact of copper on 14.89 ppm lead in 0.1 M tris–HCl buffer was next studied (Fig. 14b). The peak currents of Pb(ii) were somewhat reduced with increasing the copper concentration (14.89, 19.75, and 24.57 ppm), while the two peaks at −0.41 and −0.48 V signified lead's two-step oxidation. The linear equation for Cu(ii) in the concentration range of 14.89 to 24.57 ppm was as follows:

9 *I* (μA), Cu = 2.0413*C* (ppm) − 1.8927, (*r* = 0.955)

The HDBA/PL electrode had a higher sensitivity to Cu(ii) when lead was present (2.0413) than for copper without lead (1.9214), because Cu depressed the Pb wave and caused the formation of Cu–Pb intermetallic complexes, and so the sensitivity for copper increased while the sensitivity for lead decreased. Cu's sensitivity was increased by the oxidation of the Cu–Pb intermetallic complexes, which took place close to Cu's square potential (II).

##### 3.2.5.2 Simultaneous determination of copper and cadmium

Cadmium's effect on 14.89 ppm copper in 0.1 M tris–HCl buffer was investigated.^45^ The peak currents for Cu(ii) were slightly increased as the cadmium concentration increased (14.89, 19.75, and 24.57 ppm). The linear equation for Cd(ii) with regard to concentrations of 14.89 to 24.57 ppm was as follows:^26^

10 *I* (μA), Cd = 0.6365*C* (ppm) + 1.5144, (*r* = 0.991)

**Fig. 15:**
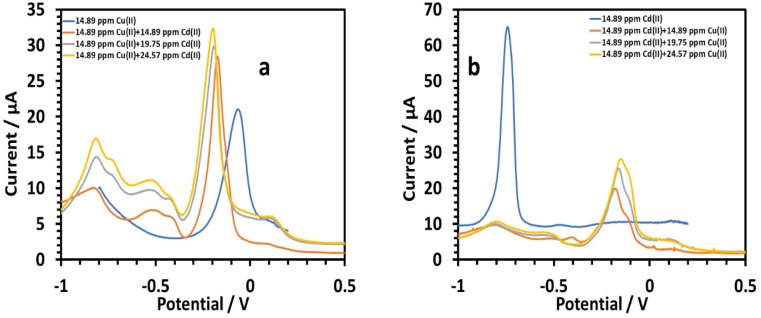
(a) Square wave voltammograms of HDBA/PL electrode solutions with different concentrations of Cd(ii) in the presence of a constant amount of Cu in 0.1 M tris–HCl buffer solution pH 6, (b) and with different concentrations of Cu(ii) in the presence of a constant amount of Cd(ii) in 0.1 M tris–HCl buffer solution pH = 5.

When copper was present, the HDBA/PL electrode had a lower sensitivity to Cd(ii). The sensitivity to cadmium without copper was 6.1955. The emergence of a wide peak in between the two main peaks (Fig. 15a) might be caused by hydrogen evolution brought on by the catalytic action; on copper electrodes, it has been demonstrated that this can happen at potentials as high as 0.40 V. The shoulder peaks around Cd could be attributed to the oxidation of Cd–Cu intermetallic complexes. Also the change in the copper peaks to more negative values was also notable. Such a peak shift is a type change of the deposited phase's signature and a marker of evolution in the Cd–Cu phase diagram. Next, the impact of copper on 14.89 ppm of cadmium in 0.1 M tris–HCl buffer was investigated. Almost no Cd(ii) stripping peaks were visible. This should be attributed to the formation of a Cd film on the surface of the modified electrode during the deposition process, since the electrode surface has a large affinity for Cd(ii), which raises Cu's sensitivity. The following is the linear equation for Cu(ii) with regard to concentrations of 14.89 to 24.57 ppm:^26^

11 *I* (μA), Cu = 0.8568*C* (ppm) + 7.6392 (*r* = 0.9599)

When cadmium was present, the HDBA/PL electrode showed lower a sensitivity to Cu(ii) of 0.8568, whereas copper's sensitivity in a cadmium-free environment was 1.9214. The oxidation of Cu–Cd intermetallic complexes was responsible for the shoulder peaks close to Cu, while the emergence of a wide peak (Fig. 15b) in between the two main peaks might be caused by hydrogen evolution brought on by copper's catalytic activity.

##### 3.2.5.3 Simultaneous determination of lead and cadmium

**Fig. 16:**
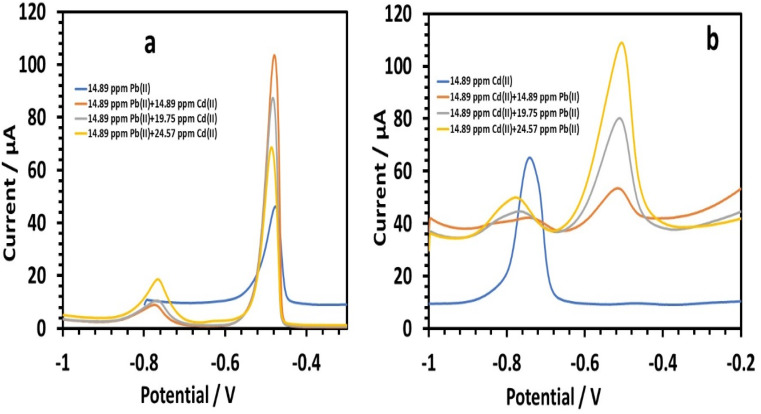
(a) Square wave voltammograms of HDBA/PL electrode solutions with different concentrations of Cd(ii) in the presence of a constant amount of Pb in 0.1 M tris–HCl buffer solution pH 3, (b) and with different concentrations of Pb(ii) in the presence of a constant amount of Cd(ii) in 0.1 M tris–HCl buffer solution pH 5.

Next, we investigated the impact of cadmium on 14.89 ppm lead in 0.1 M tris–HCl buffer. Varying the amounts of cadmium (14.89, 19.75, and 24.57 ppm) marginally increased the peak currents of Pb(ii) (Fig. 16a). The following is the linear equation for Cd(ii) with regard to concentrations of 14.89 to 24.57 ppm:^26^

12 *I* (μA), Cd = 0.9738*C* (ppm) − 6.507, (*r* = 0.8601)

When there was lead present, the HDBA/PL electrode showed a lower sensitivity to Cd(ii). The sensitivity of cadmium without lead was 6.1955 *versus* 0.9738 with lead present. We investigated the impact of lead on 0.1 M tris–HCl buffer containing 14.89 ppm cadmium, with lead concentrations of 14.89, 19.75, and 24.57 ppm (Fig. 16b), and found the Cd(ii) peak currents were only slightly diminished. The following is the linear equation with regard to concentrations of 14.89 to 24.57 ppm for Pb(ii):^26^

13 *I* (μA), Pb = 5.7362*C* (ppm) − 32.328, (*r* = 0.9994)

The HDBA/PL electrode had a greater Pb(ii) sensitivity when cadmium was present (5.7362), compared to without cadmium (2.7689). The cadmium peak was tilted more in the direction of negative values. Peak shifting is a type of change in the deposited phase's hallmark that denotes a progression in the Cd–Cu phase diagram. The effect of Cd on Pb(ii) sensitivity was essentially nonexistent. Since there were no shoulder peaks, it could be concluded that the peak suppression of Cd by Pb was not caused by the intermetallic interference between Cd and Pb.^46^ Rather, the increased diffusivity and higher standard reduction potential of Pb(ii) may be the cause.

#### 3.2.6. Selectivity of the HDBA/PL electrode

**Fig. 17:**
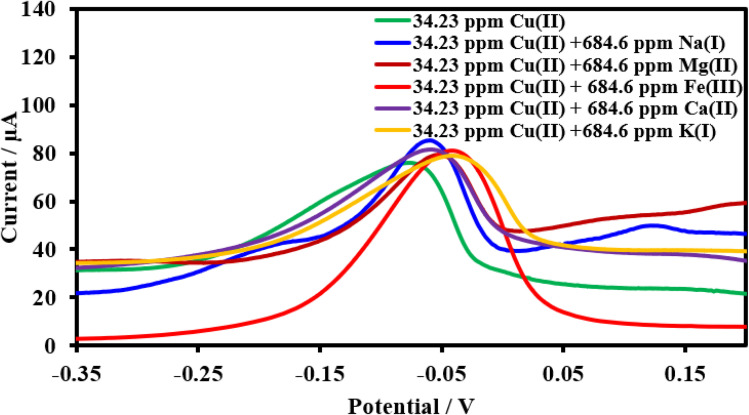
Square wave voltammograms of the HDBA/PL electrode with 34.23 ppm Cu(ii), pH 6 in the absence and presence of the interfering metal ions Na^1+^, Mg^2+^, Fe^2+^, Ca^2+^, and K^1+^.

**Fig. 18:**
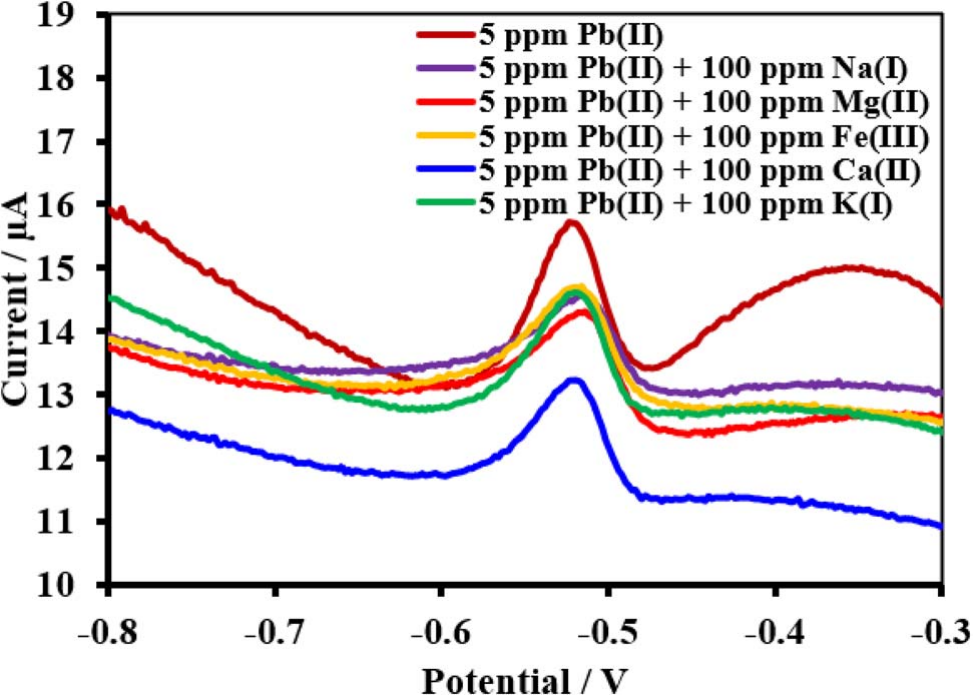
Square wave voltammograms of the HDBA/PL electrode with 34.23 ppm Pb(ii), pH 3 in the absence and presence of the interfering metal ions Na^1+^, Mg^2+^, Fe^2+^, Ca^2+^, and K^1+^.

**Fig. 19:**
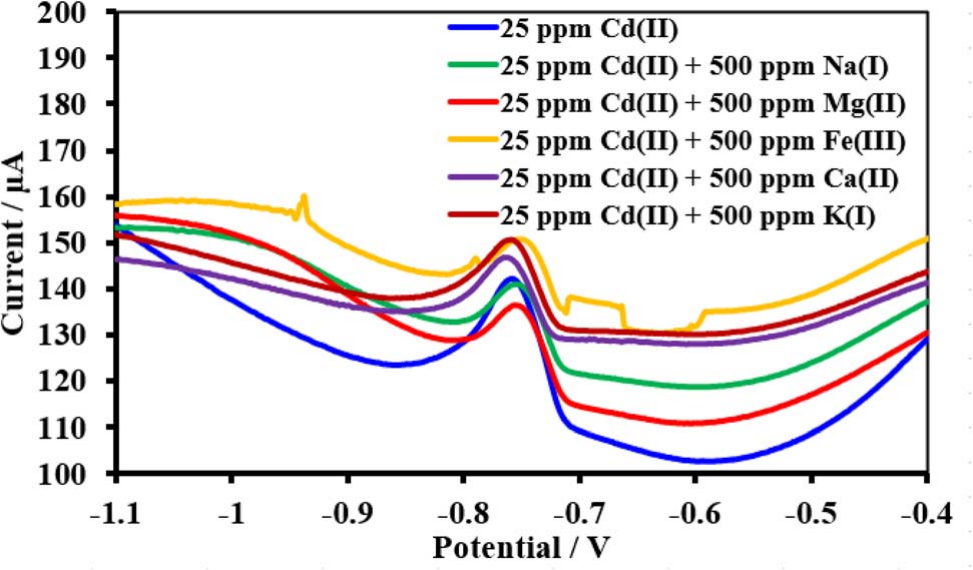
Square wave voltammograms of the HDBA/PL electrode with 34.23 ppm Cd(ii), pH 5 in the absence and presence of the interfering metal ions Na^1+^, Mg^2+^, Fe^2+^, Ca^2+^, and K^1+^.

Under pH-optimized circumstances, interference analysis was carried out by detecting 34.23 ppm Cu(ii), 5 ppm Pb(ii), and 25 ppm Cd(ii) in the presence of 20-fold concentrations of interfering ions (Fig. 17–19). The peak currents of Cu(ii), Pb(ii), and Cd(ii) were often changed by less than 10%, as shown in Table 4, while the peak currents of Cu(ii) increased by 12.2% with Na^1+^ and the peak current of Pb(ii) decreased by 15.9% with Ca^2+^. It is likely that the excessive Na^1+^, Ca^2+^, and target metals on the electrode surface may be competing with one another, which might be the cause for these results. In the SWV tests, the HDBA/PL electrode showed good selectivity for Cu(ii), Pb(ii), and Cd(ii).^47,48^

**Table 4:** Interferences of some metal ions on SWV analysis of the peak current of Cu(ii), Pb(ii), and Cd(ii) with the HDBA/PL electrode

Interferences	Peak current change of Cu(ii) (%)	Peak current change of Pb(ii) (%)	Peak current change of Cd(ii) (%)
Na^1+^	+12.2	−7.3	−0.9
Mg^2+^	+4.5	−9.0	−4.0
Fe^+3^	+7.0	−6.4	+5.9
Ca^2+^	+7.3	−15.9	+3.0
K^1+^	+4.1	−6.9	+5.9

## 4. Conclusion

This article looked into the specific electrochemical behavior of Cu, Pb, and Cd using a simple HDBA/PL electrode. The described modified electrode greatly enhanced the electrochemistry of the metals and amply illustrated the remarkable HDBA/PL electrode's activity in terms of the anodic response to heavy metals. The electrochemical properties were computed for the apparent charge transfer rate constant, transfer coefficient, diffusion coefficient, and surface concentration of the electroactive species. Satisfactory analytical performance was obtained under the optimized experimental circumstances, including an adequate precision. The technique was efficient enough to analyze heavy metal contents at lower levels. Additionally, the suggested approach does not call for pricey equipment or essential analytical reagents.

## Conflicts of interest

The authors declare that they have no known competing financial interests or personal relationships that could have appeared to influence the work reported in this paper.

## Supplementary Material
